# Cortical, subcortical, and cerebellar atrophy and cognition deficits in Metropolitan Mexico City teens and young adults exposed to fine particulate matter (PM_2.5_) - neurodegeneration is in progress

**DOI:** 10.3389/fneur.2026.1772916

**Published:** 2026-03-24

**Authors:** Lilian Calderón-Garcidueñas, Jacqueline Hernández-Luna, Carolina I. Galaz-Montoya, Sean A. P. Clouston, Mario Vincenzo Aiello-Mora, Julio Amaro de Gante, Elijah W. Stommel, Ricardo Torres-Jardón, Ozkan Ufuk Nalbantoglu

**Affiliations:** 1 The University of Montana, Missoula, MT, United States; 2 Radiology Department, Hospital HMG, Coyoacán, Mexico City, Mexico; 3 Departments of Child Health, Neurology, and Cellular & Molecular Medicine, and Program in Genetics, Phoenix, AZ, United States; 4 Genetics, GIDP PhD Program, Tucson, AZ, United States; 5 Pediatric Movement Disorders Program, Division of Pediatric Neurology, Barrow Neurological Institute, Phoenix Children’s Hospital, Phoenix, AZ, United States; 6 Department of Family, Population, and Preventive Medicine, Renaissance School of Medicine at Stony Brook University, Stony Brook, NY, United States; 7 Servicio de Otorrinolaringología, Instituto Nacional de Cardiología Ignacio Chávez, Mexico City, Mexico; 8 Department of Neurology, Geisel School of Medicine, Dartmouth-Hitchcock Medical Center, Lebanon, NH, United States; 9 Instituto de Ciencias de la Atmósfera y Cambio Climático, Universidad Nacional Autónoma de México, Mexico City, Mexico; 10 Department of Computer Engineering, Dokuz Eylul University, İzmir, Türkiye

**Keywords:** adolescents and young urbanites brain atrophy, air pollution, Alzheimer, Parkinson and TDP43 pathology in children, brain MRI in urbanites early diagnosis neurodegeneration, fine particulates PM_2.5_, overlapping neurodegeneration in children and young adults, subcortical and cerebellar atrophy

## Abstract

Exposure to environmental fine particulate matter (PM_2.5_), ultrafine PM (UFPM) and nanoparticles (NPs) are associated with accumulation of amyloid-β_1–42_ peptides, phosphorylated-Tau, alpha-synuclein and transactive response DNA binding-protein-43 misfolded aberrant proteins, consistent with the biological definitions of overlapping Alzheimer’s disease (AD), Parkinson’s disease (PD), frontotemporal lobar degeneration (FTLD), and amyotrophic lateral sclerosis (ALS) in 99% of ≤40-year-old Metropolitan Mexico City (MMC) forensic autopsies. Structural and volumetric brain responses *in vivo* are critical in young MMC residents. We performed volumetric and whole-brain correlation analyses in 75 healthy volunteers: 45 MMC 31.2 ± 14.7 y old and 30 low-pollution 31.8 ± 4.8 y old controls, matched by ethnicity, socioeconomic status, nutrition, and BMI. MMC residents exhibited fronto-parietal and temporal lobes, precentral gyrus, hippocampi, basal ganglia, thalamus, amygdala and cerebellar atrophy. The most common atrophy pattern was *cortical* first parietal and fronto-parietal lobes, combined with gray matter (GM) atrophy in cerebellar lobules IV and V left and right III, IV and V and VI.MMC participants had mild cognitive impairment (Montreal Cognitive Assessment Score 22.8 ± 3.2). GM atrophy involving right globus pallidus and pulvinar and cerebellar white matter (WM) bilaterally were associated with lower cognitive performance and high BMI to subiculum, posterior orbital gyrus and insula, inferior temporal gyrus, supplementary motor cortex, and cuneus WM atrophy. PM_2.5_ exposure and BMI appear to play key roles in early neurodegenerative disease biology and may contribute to adverse effects on academic and occupational performance, neuropsychiatric disorders, behavioral regulation, risk of substance use initiation, and psychopathy. Neuroradiologists across the world need to know cortical and subcortical, including extensive hippocampal, stratium and cerebellar atrophy identifies overlapping patterns of regional atrophy associated with MCI, AD, bvFTD, PD and ALS, in young urbanites. There is an urgent need for early pediatric neuroprevention interventions, non-invasive AD, PD and TDP-43 biomarkers, in-depth characterization of emission pollutants exposures and their effective control. Denial is no longer an option.

## 1 Introduction

Metropolitan Mexico City (MMC) residents have been exposed to fine particulate matter PM_2.5_ concentrations above the current United States Environmental Protection Agency (US EPA) annual standard of 9 μg/m^3^ for decades, starting in utero (1–6). Young MMC residents, including toddlers, exhibit supra- and infra-tentorial aberrant hyperphosphorylated tau (p-*τ*), β-amyloid, α-synuclein, and transactive response DNA-binding protein 43 (TDP-43) pathology associated with highly reactive, magnetic ultrafine particulate matter (UFPM), and industrial nanoparticles (NPs) (7–10).

In MMC vs. low-pollution matched children aged 8.8 ± 1.65 years, fluid and crystallized cognition deficits were observed along with MRI prefrontal white matter hyperintense lesions, WM volume differences in bitemporal and right parietal lobes, while the hippocampus, caudate, putamen, globus pallidus, and amygdala exhibited no volumetric differences between cohorts (11, 12). In 302 MMC volunteers (mean age 32.7 ± 6.0 years) with annual residential PM_2.5_ exposures of 27.6 ± 1.6 μg/m^3^, hemispheric volumetric differences in the frontal and temporal lobes, caudate, and cerebellar gray matter (GM) and WM were observed when comparing ≤30-year-old versus ≥31-year-old MMC residents (13). Significant associations were identified between Montreal Cognitive Assessment (MoCA) total and index scores and bilateral caudate atrophy, frontal and temporal atrophy, and cerebellar WM reductions (13).

Cognition deficits are a consistent finding in prior work, MoCA language index scores (animal naming, sentence repetition, and word fluency) were early targets, and MMC residency was associated with multi-domain cognitive impairment and structural brain atrophy patterns described in Alzheimer’s disease (AD), Parkinson’s disease (PD), and frontotemporal lobar degeneration (FTLD) (13, 14).

To date, *in vivo* imaging of neurodegenerative characteristics in children and young adult MMC residents is not well established.

We aimed to address two unresolved questions in MMC versus low-pollution healthy, young, middle-class, mostly college-educated volunteers: (1) to measure cortical surface area and thickness, subcortical and cerebellar volumes using T1-weighted MR images to identify regional brain targets; and (2) to analyze MoCA and body mass index (BMI) results and their associations with MRI findings to identify subjects at highest risk. We hypothesized that lifetime PM_2.5_ exposures above the current US EPA annual standard (1) would be associated with neurodegeneration on neuroimaging and reduced cognitive performance.

MMC young residents exhibit significant brain atrophy and strong correlations between MoCA scores and BMI, affecting targeted cortical and subcortical brain regions. These findings in clinically healthy young urban residents have early potential value as non-invasive tools for identifying early neurodegenerative changes.

## 2 Materials and methods

### 2.1 Study population, inclusion, and exclusion criteria

This prospective 2022–2024 protocol was approved by the review boards and ethics committees at the University of Montana (IRB# 206R-09 and 185-20). Written consent was obtained from the adult volunteers and from parents of minors, who also signed a verbal consent.

The participating 75 volunteers included: 1. 45 MMC subjects with brain MRI/MoCA age 31.2 ± 14.7, including 5 children aged 15 ± 2 years and 2. 30 controls, with PM_2.5_ exposures below the annual USEPA standard, 31.8 ± 4.8 years. Subjects matched by ethnicity, socioeconomic status, nutrition patterns, and years of formal education were considered clinically healthy. Exclusion criteria included a family history of neurodegenerative diseases (i.e., AD, PD, FTLD, and ALS), participation in contact sports, head trauma, occupational or household exposures to toxic substances, and a history of cannabis use or illegal drug use. Occasional social drinking was recorded in 11% of volunteers. All volunteers lived in their respective cities from birth, and their mothers got pregnant while living in the targeted city. Moreover, they lived in the same area their entire life. Each volunteer’s permanent residence address was recorded.

### 2.2 Brain MRI acquisition and processing

Three-dimensional MRI for all subjects was acquired on a 1.5 Tesla 5T Signa Excite HD MR (General Electric, Milwaukee, WI, United States) with an 8-channel brain array coil (13). CAT12 was used to measure sub-anatomical volumes and cortical thickness in study participants using cropped imaging databases. Data were segmented into anatomical components using the standard segmentation pipeline (15, 16). For region-of-interest analyses, we output anatomical features for cortical and subcortical structures and measure hippocampal, amygdala, and cerebellar volumes (15–17).

### 2.3 Neurocognitive performance

The Spanish version of the Montreal Cognitive Assessment (MoCA) is a brief tool designed to identify and characterize individuals with mild to moderate cognitive impairment (18).

### 2.4 Covariates

Demographic variables included age, sex, years of formal education, body mass index (BMI; kg/m^2^), and MoCA total score. The intracranial volume (ICV) was also estimated.

### 2.5 Study city and air quality

#### 2.5.1 Metropolitan Mexico City (MMC)

MMC, with approximately 24 million residents, is located in central Mexico in a semi-enclosed basin of approximately 23,000 km^2^ at an average elevation of 2,200 m above sea level. Approximately 75,000 industries and 6 million vehicles consume more than 175 million liters of petroleum-derived fuels per day, including natural and liquefied petroleum (LP) gas.

Vehicular emissions dominate the MMC inventory, with older heavy-duty diesel vehicles accounting for more than 30% of total PM_2.5_ emissions. Particle-bound polycyclic aromatic hydrocarbons (PAHs) represent 30–50% of their mass. PM_1_ (particles with a diameter <1 μm) represents approximately 70% of PM_2.5_ mass. Public transport use and walking along sidewalks in MMC are associated with high nanoparticle (NPs ≤ 100 nm) exposures (19, 20).

Pedestrians on high-traffic avenues are exposed to NPs measuring 49 ± 20 nm, with Fe, Pb, and Zn composing approximately 60%. The subway system presents elevated exposure levels, with PM_2.5_ concentrations ranging from 34 to 93 μg/m^3^, PAHs between 19 and 41 ng/m^3^, and NP number concentrations of 50,300 ± 10,600 particles/cm^3^ (38.5 ± 15.9 nm), composed of Fe, Cu, Ni, Cr, and Mn. Metals present in the underground subway result from friction, brake wear, and sparking from rail grinding (2, 3, 19, 20).

The combination of vehicular and industrial gas-phase pollutants (NOx emissions), volatile organic compounds (VOCs), household solvents, LP gas leaks, oil and gasoline vapors, strong solar radiation, and poor basin ventilation results in high ozone levels and secondary particle formation (2).

#### 2.5.2 Control city: Hermosillo

Hermosillo, with ~940,000 people, good wind ventilation, is located at the southern extreme of the Sonoran Desert in northwestern Mexico. Hermosillo has concentrations of PM_2.5_, SO_2_, NO_2_, CO, SO_2_, and O_3_ below the respective US EPA National Ambient Air Quality Standard (NAAQS) for short- and long-term exposures (21).

### 2.6 Statistical analysis

Continuous variables were summarized using means and standard deviations. Multiple linear regression analyses were performed to test the significance of mean group differences after adjusting for age, sex, BMI, and years of education, comparing (i) highly exposed MMC volunteers and (ii) low-pollution controls.

For each regional brain volume or cortical thickness value, the regression model included group status together with the selected covariates. Statistically significant results were mapped onto the cerebral surface using a heat-mapping approach, with “hot” colors to visualize cortical atrophy distribution.

Adolescents and young adults aged ≤20 years with high MMC exposure were compared with matched lower-exposure individuals by sex and age. Multivariate linear regression analyses were also performed to evaluate associations between BMI, MoCA scores, regional cerebral volume, cortical thickness, age, and intracranial volume.

## 3 Results

### 3.1 Air pollution

**Figure 1:**
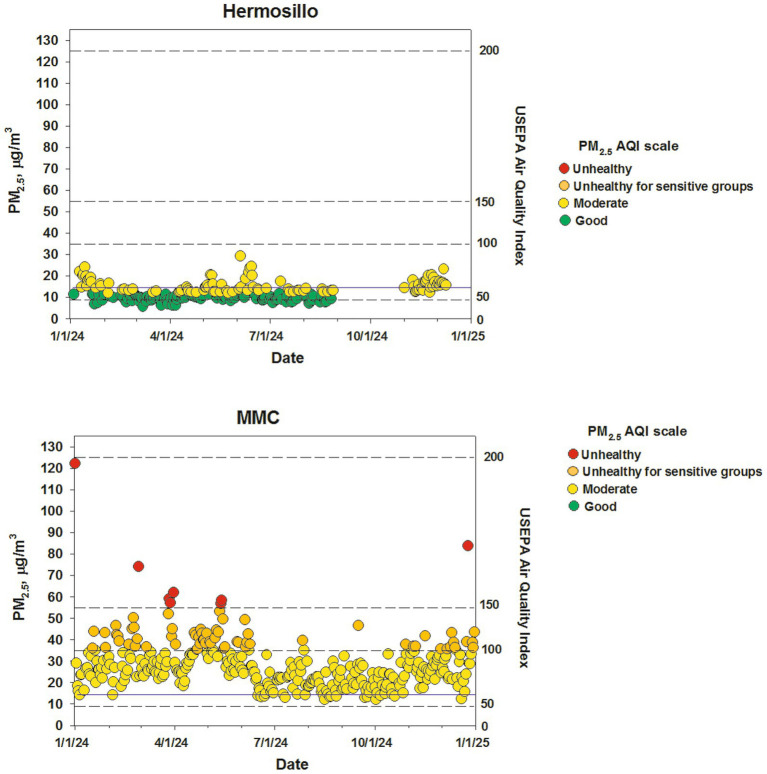
Time series of PM_2.5_ 24-h averages for Hermosillo and MMC, 1 January to 31 December 2024 (US EPA AQI index).

MMC residents have been exposed to PM_2.5_ above the annual US EPA NAAQS for the past three decades. The 24-h PM2.5 time series for MMC and Hermosillo in 2024 are shown in Figure 1. Hermosillo had daily mean PM_2.5_ concentrations below the US EPA Air Quality Index (AQI) standard, whereas PM_2.5_ levels in MMC exceeded the AQI level of 100.

### 3.2 Study population and demographics

Table 1 summarizes the demographics of the 75 participating volunteers, including 30 control residents.

**Table 1:** Summary of age, BMI, years of formal education, and MoCA scores among 75 volunteers, including controls.

Residency	Average age (years)	MoCA score	Education (years)	BMI (kg/m^2^)
All MMC (*n* = 45)	31.2 ± 14.7	22.8 ± 3.2	13.4 ± 4.6	24.2 ± 3.4
Clean air controls (*n* = 30)	31.8 ± 4.8	26.2 ± 1.2	18.5 ± 2.2	24.3 ± 2.5

### 3.3 MMC *versus* controls: cortical thickness, regional GM and WM volumes, cortical surface area, and ICV

We examined differences in cortical thickness and identified a significant global difference in cerebral GM (*p* = 0.0054). Regional differences were then evaluated in subregional analyses (Table 2).

**Table 2:** *Q*-values rank-ordered statistical significance in cortical thickness in highly exposed MMC volunteers versus controls.

Cortical thickness (mm)	Q-Value
Temporal, superior, left	0.002
Temporal, superior, right	0.002
Frontal, caudal middle, right	0.005
Parietal, inferior, right	0.005
Cingulate, posterior, right	0.005
Parietal, inferior, left	0.005
Occipital, lateral, right	0.005
Paracentral lobule, left	0.005
Postcentral gyrus, left	0.005
Frontal, caudal middle, left	0.005
Precuneus, right	0.005
Frontal, rostral middle, right	0.005
Paracentral lobule, right	0.005
Occipital, lateral, left	0.007
Precuneus, left	0.006
Precentral, right	0.006
Frontal, superior, left	0.006
Frontal, superior, right	0.006
Supramarginal, right	0.006
Cuneus, left	0.007
Supramarginal, left	0.007
Cuneus, right	0.009
Precentral, left	0.010
Pars opercularis, left	0.011
Rostral middle frontal, left	0.012
Pars triangularis, right	0.025
Pars orbitalis, right	0.029

Cortical GM was significantly decreased in MMC residents, predominantly in the left hemisphere, including the superior temporal gyrus, superior, middle, and inferior frontal lobes, precentral gyrus (primary motor cortex), inferior parietal lobe, and lateral occipital regions. Robust bilateral results findings were identified in the superior temporal lobes (*Q* = 0.002).

We next examined overall volume changes in subcortical GM regions, including the basal ganglia, hippocampus, thalamus, and amygdala (Table 3).

**Table 3:** Volumetric studies of the hippocampus, striatum, thalamus, and amygdala in MMC versus controls, rank-ordered by *Q*-value.

Parameter	*Q*-value
Pallidum, gray matter, left	5.60 × 10^−14^
Globus pallidus, white matter, left	2.10 × 10^−13^
Thalamus, gray matter, right	2.10 × 10^−13^
Alveus, gray matter, right	2.25 × 10^−06^
Hippocampus, gray matter, left	3.30 × 10^−06^
Fimbria, gray matter, left	5.98 × 10^−06^
Globus pallidus, white matter, right	2.45 × 10^−05^
Pallidum, gray matter, right	4.43 × 10^−05^
Striatum, white matter, right	5.90 × 10^−05^
Fimbria, gray matter, right	6.19 × 10^−05^
Alveus, gray matter, left	1.01 × 10^−04^
Hippocampus, gray matter, CA1/2 left	1.76 × 10^−04^
Hippocampus, gray matter, CA4, right	2.07 × 10^−04^
Hippocampus, gray matter, right	4.80 × 10^−04^
Hippocampus, gray matter, CA4, left	1.43 × 10^−03^
Hippocampus, gray matter, CA1/2, right	1.43 × 10^−03^
Amygdala, gray matter, right	4.64 × 10^−03^
Globus pallidus, gray matter, right	7.52 × 10^−03^
Putamen, gray matter, right	1.11 × 10^−02^
Putamen, gray matter, left	1.78 × 10^−02^
Striatum, gray matter, left	1.97 × 10^−02^
Caudate, gray matter, right	3.23 × 10^−02^
Striatum, gray matter, right	4.65 × 10^−02^

Atrophy in MMC subjects included the whole hippocampus and the CA1/2 and CA4 subregions, as well as bilateral putamen, globus pallidus, pallidum, alveus, and fimbria. Right-dominant differences were identified in the caudate, amygdala, thalamus, and globus pallidus GM. Atrophy was highly significant in the pallidum, globus pallidus, and thalamus (*Q* < 1 × 10^−7^).

Strikingly, cerebellar GM and WM atrophy in MMC were significant and widespread (Table 4). Nearly all cerebellar WM and GM regions differed statistically between MMC and controls.

**Table 4:** Differences in cerebellar volume in MMC versus controls, rank-ordered by *Q*-value.

Parameter	*Q*-value
White matter, right, lobule VI	4.97 × 10^−17^
White matter, superior posterior, right, lobule VI	1.04 × 10^−14^
White matter, exterior, right	5.60 × 10^−14^
White matter, left, lobule VI	2.10 × 10^−13^
White matter, left, lobule V	4.87 × 10^−13^
White matter, exterior, right	7.39 × 10^−12^
White matter, anterior, right, lobule V	1.93 × 10^−11^
White matter, right, lobule V	1.88 × 10^−10^
White matter, left, lobule V	2.47 × 10^−10^
White matter, right, lobule VIIIA	1.42 × 10^−09^
White matter, left, Crus I	8.79 × 10^−09^
White matter, right, inferior posterior, lobule VIIIA	1.04 × 10^−08^
White matter, left, lobule IV	1.57 × 10^−08^
White matter, right, Crus I	2.64 × 10^−08^
White matter, left, lobule VIIB	3.94 × 10^−08^
White matter, inferior posterior, left, lobule VIIIA	3.94 × 10^−08^
White matter, anterior, right, lobule IV	4.32 × 10^−08^
White matter, left, lobule VIIB	4.69 × 10^−08^
White matter, left, lobule VIIIA	1.67 × 10^−07^
White matter, right, lobule IV	1.69 × 10^−07^
White matter, left, lobule IV	2.30 × 10^−07^
White matter, left, Crus II	2.30 × 10^−07^
White matter, right, lobule VIIB	2.30 × 10^−07^
Gray matter, left, lobule V	4.55 × 10^−07^
White matter, right, lobule VIIB	6.52 × 10^−07^
Gray matter, right, lobule V	1.18 × 10^−06^
Gray matter, right, lobule IV	3.02 × 10^−06^
Gray matter, right, lobule V	3.02 × 10^−06^
Gray matter, left, lobule VI	8.74 × 10^−06^
Gray matter, superior posterior, left, lobule VI	1.06 × 10^−05^
Gray matter, left, lobule V	1.16 × 10^−05^
Gray matter, right, sup post, lobule VI	4.66 × 10^−05^
White matter, right, Crus II	5.20 × 10^−05^
Gray matter, right, lobule VI	5.35 × 10^−05^
White matter, right, inferior post, lobule VIIIB	6.48 × 10^−05^
Gray matter, left, lobule IV	7.90 × 10^−05^
Vermis, Crus II	3 × 10^−04^
Gray matter, right, lobule IV	5 × 10^−04^
Gray matter, left, lobule V	6 × 10^−04^
Cerebellar, vermal lobules VIII–X	9 × 10^−04^
White matter, CSF, left	9 × 10^−04^
White matter, left, lobule VIIIB	1 × 10^−03^
Cerebellum, exterior, CSF, left	2 × 10^−03^
White matter, inferior posterior, left, lobule VIIIB	3 × 10^−03^
White matter, CSF, right	6 × 10^−03^
Gray matter, left, lobule I/II	7 × 10^−03^
Gray matter, right, lobule X	7 × 10^−03^
White matter, right, lobule III	8 × 10^−03^
Vermis, lobules VIII–X	8 × 10^−03^
Gray matter, right, lobule III	1.50 × 10^−02^
Cerebellum, exterior, CSF, right	2.60 × 10^−02^
Gray matter, left, lobule III	3.50 × 10^−02^

**Figure 2:**
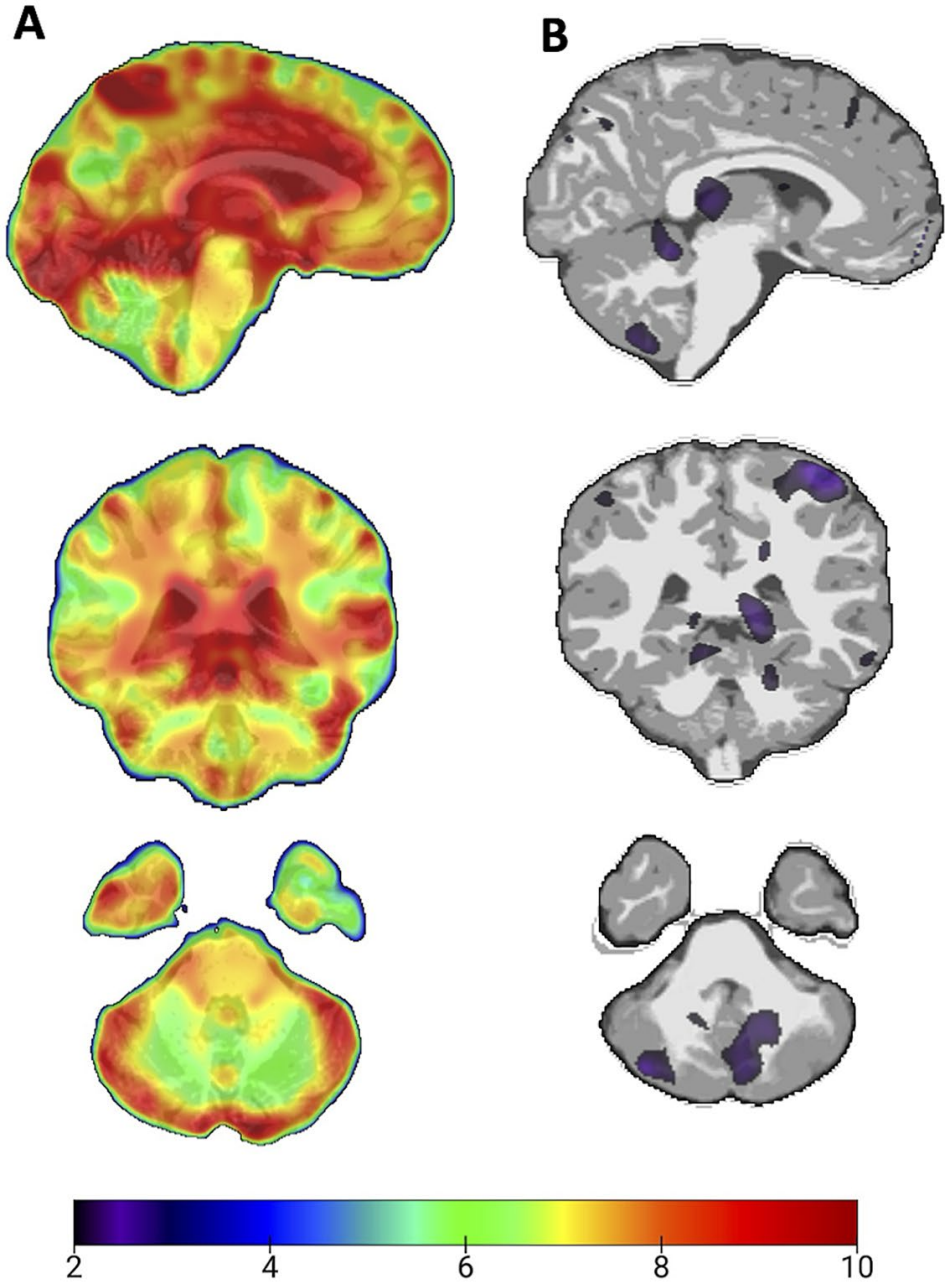
Volumetric outcomes across the entire brain in females aged 20–25 years, varying in exposure. **(A)** Highly exposed MMC participant. **(B)** Low-exposure participant. Colors are shown if they exceed an intensity threshold (≥2) and range from blue (mild increase) to red (high increase). The control shows predominantly black/white values. On the left, an MMC female shows extensive areas of atrophy, including the hemispheric cortex and cerebellum.

Threshold-based case-specific results comparing individuals in MMC vs. controls on volumetric outcomes across the entire brain, as determined by deformation-based morphometry relative to Diffeomorphic Anatomical Registration Through Exponentiated Lie Algebra (DARTEL) standards in (A) MMC and (B) control females aged 20–25 years, as shown in Figure 2.

Widespread degeneration across the entire brain was evident in MMC individuals, as shown by the rainbow heatmap (Figure 2). In contrast, low-exposure individuals had values predominantly below threshold, although limited regions showed mild deviations relative to DARTEL healthy reference data (Figure 2).

**Figure 3:**
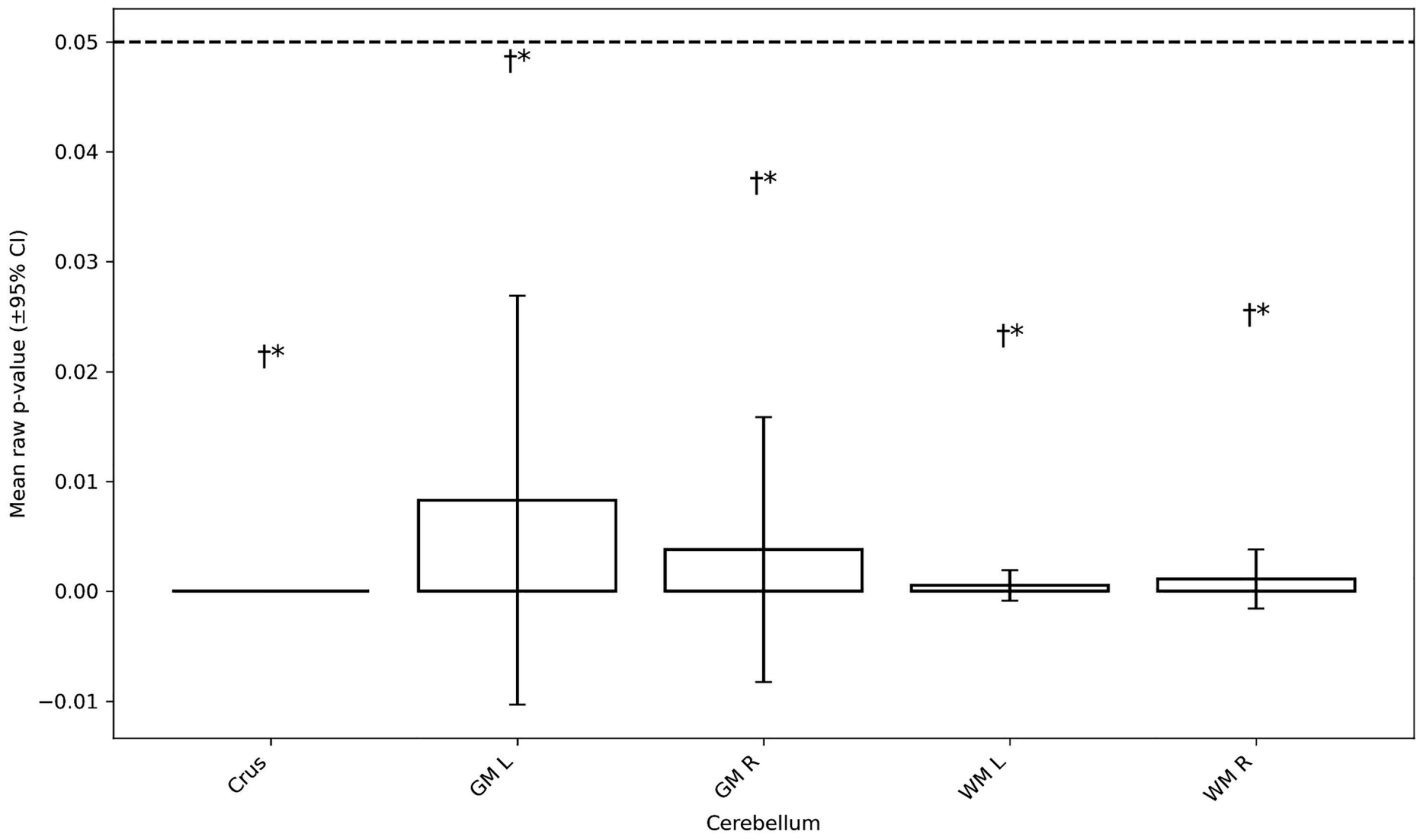
Mean *p*-values for group differences by cerebellar subregion [±95% confidence interval (CI)]. Hollow bars show mean *p*-values for group comparisons across five cerebellar subregions: crus, GM left, GM right, WM left, and WM right. Error bars represent two-sided, *t*-based 95% CIs; the dashed line marks *p* = 0.05. ^†^Indicates that the entire region had *p* < 0.05; ^*^indicates that at least one test in that subregion survived Benjamini–Hochberg FDR at *α* = 0.05.

Figure 3 shows extensive cerebellar GM and WM involvement in MMC vs. controls, including the cerebellar vermis and hemispheric lobules.

**Figure 4:**
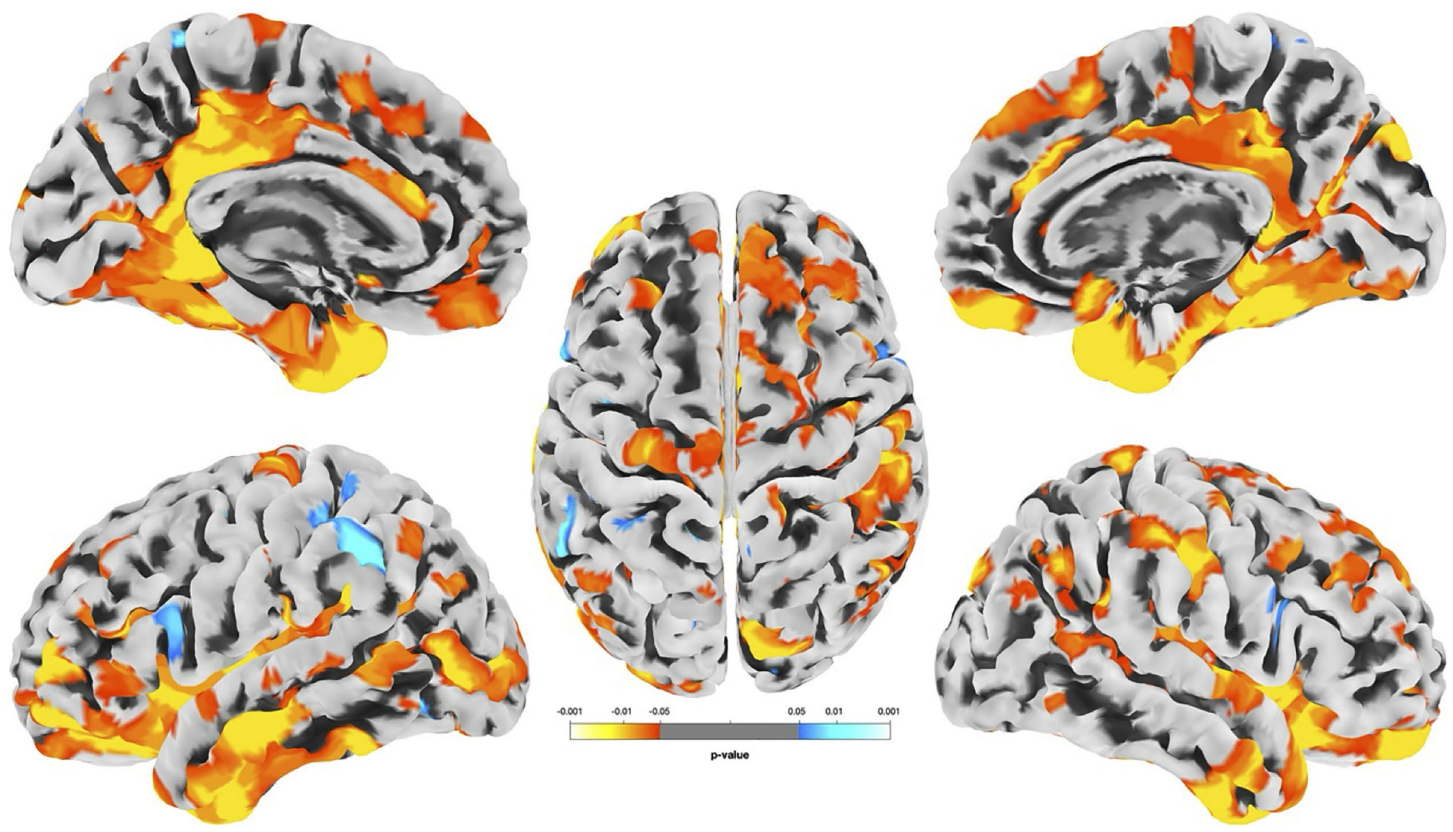
Groupwise differences in cortical thickness across the cerebral surface (*p* < 0.05). Decreases (blue hues) and increases (red hues) are shown. Results using threshold-free cluster enhancement revealed that all colored regions passed nominal two-tailed *α* = 0.05, while clusters highlighted in yellow survived false discovery rate correction (FDR = 0.05). Peaks were located in the lingual gyrus, bilateral temporal poles, right posterior cingulate, fusiform gyrus, right inferior parietal and lateral occipital, left postcentral gyrus, and right precentral gyrus.

Replicating findings in the full cohort but focusing on MMC adolescents, groupwise vertex-wise differences in cortical thickness between high- and lower-exposed adolescents were identified (FDR = 0.05) (Figure 4).

These results revealed large statistically significant clusters (highlighted in yellow). Cluster peaks where effect sizes appeared strongest were located in the lingual gyrus, bilateral temporal pole, isthmus cingulate, fusiform gyrus, right inferior parietal and lateral occipital, left postcentral gyrus, and right precentral gyrus.

### 3.4 MoCA and MRI correlations: MMC versus controls

MMC residents had a mean MoCA score of 22.8 ± 3.2, within the mild-to-moderate cognitive impairment range, compared with controls, who had 26.3 ± 1.3 (Table 1). Linear regression of MoCA scores in MMC participants aged 20–40 years showed a slope of −0.147 MoCA points per year (95% CI: −0.225, −0.068; *p* = 0.000346), corresponding to an average decline of 2.93 MoCA points over 20 years (95% CI: −4.51, −1.35).

MoCA scores showed positive associations with bilateral pallidal GM, right pulvinar GM, and bilateral cerebellar WM (including lobules VI, VIIB, and VIIIA). Higher MoCA scores were associated with larger volumes in these regions. Lower MoCA scores were associated with atrophy in the right globus pallidus GM (*β* = +0.00137, FDR = 1.33 × 10^−4^), right pulvinar GM (*β* = +0.00147, FDR = 0.0187), and bilateral cerebellar WM (*β* ≈ +0.018–0.019, FDR ≈ 0.022–0.023).

### 3.5 BMI and MRI results

We analyzed correlations between BMI and cortical and subcortical volumes in MMC versus controls (Table 5).

**Table 5:** Differences in cortical and subcortical volume MMC versus controls, BMI, rank-ordered by *p*-value.

Volume	*p*-value
Subiculum, white matter, right_ptiv	4.20 × 10^−5^
Orbital gyrus, posterior, white matter, right_ptiv	7.69 × 10^−4^
Frontal pole, white matter, left_ptiv	1.59 × 10^−3^
Insula, posterior, white matter, left_ptiv	3.43 × 10^−3^
Insula, posterior, white matter, right_ptiv	3.53 × 10^−3^
Temporal gyrus, inferior, white matter, right_ptiv	3.55 × 10^−3^
Occipital, fusiform gyrus, white matter, right_ptiv	5.20 × 10^−3^
Subiculum, white matter, left_ptiv	5.76 × 10^−3^
Lingual gyrus, white matter, left_ptiv	6.83 × 10^−3^
Hippocampus, white matter, right_ptiv	8.79 × 10^−3^
Occipital inferior gyrus, white matter, right_ptiv	1.00 × 10^−2^
Calcarine cortex, white matter, left_ptiv	1.30 × 10^−2^
Temporal superior gyrus, gray matter, left_ptiv	1.39 × 10^−2^
Cuneus, white matter, right_ptiv	1.40 × 10^−2^
Cuneus, white matter, left_ptiv	1.41 × 10^−2^
Superior frontal gyrus, medial segment, left_ptiv	1.42 × 10^−2^
Supplementary motor cortex, white matter, right_ptiv	1.46 × 10^−2^
Fornix, white matter, left_ptiv	1.81 × 10^−2^
Lingual gyrus, white matter, right_ptiv	1.94 × 10^−2^
Calcarine cortex, white matter, right_ptiv	1.97 × 10^−2^
Frontal superior gyrus, white matter, right_ptiv	2.11 × 10^−2^
Inferior Frontal gyrus, triangular part, WM, left_ptiv	2.25 × 10^−2^
Precentral gyrus, gray matter, left_ptiv	2.68 × 10^−2^
Supramarginal gyrus, gray matter, right_ptiv	2.79 × 10^−2^
Midorbital gyrus, white matter, right_ptiv	2.82 × 10^−2^
Precuneus, white matter, left_ptiv	2.94 × 10^−2^
Temporal gyrus, inferior, white matter, left_ptiv	2.95 × 10^−2^
Cerebellum, lobule X, white matter, left_ptiv	3.29 × 10^−2^
Striatum, gray matter, left_ptiv	3.38 × 10^−2^
Precuneus, white matter, right_ptiv	3.63 × 10^−2^
Supramarginal gyrus, gray matter, left_ptiv	3.64 × 10^−2^
Cerebellum, inferior post lobule VIIIa, gray matter, right_ptiv	3.99 × 10^−2^

Higher BMI was significantly associated with WM atrophy in MMC involving the right subiculum, posterior orbital gyrus, posterior insula, inferior temporal gyrus, fusiform gyrus, inferior occipital gyrus, supplementary motor cortex, and cuneus. Significant associations were also observed in the left WM frontal pole, inferior frontal gyrus, inferior temporal gyrus, precuneus, fornix, insula, subiculum, lingual gyrus, calcarine cortex, and cerebellar lobule X.

GM atrophy associated with higher BMI was documented in the left superior temporal gyrus, precentral gyrus, and striatum, while on the right-side involvement was observed in the cerebellar lobule VIIIa and the supramarginal gyrus.

## 4 Discussion

Lifelong exposures to PM_2.5_, within complex mixtures of outdoor, indoor, and occupational air pollutants, are associated with statistically significant brain atrophy and cognition deficits in young megacity urbanites without comorbidities or a family history of neurodegenerative disease, compared with clean-air controls.

Cortical and subcortical atrophy, including extensive hippocampal, striatal, and cerebellar involvement, identifies overlapping regional atrophy patterns associated with mild cognitive impairment (MCI), AD, behavioral variant frontotemporal dementia (bvFTD), and PD (22–33). A major concern is the documentation of brain MRI alterations and cognitive deficits in the majority of young, seemingly healthy, middle-class MMC residents, and the fact that their neuroimaging findings coincide with the documented neurodegeneration continuum observed in forensic MMC autopsies (7–10). Atrophy involving the precuneus, cingulate cortex, hippocampus, temporal lobes, frontal regions, and inferior parietal lobules likely contributes to impairments in memory, executive function, emotional regulation, behavior, and social interactions and has been documented on the AD continuum (22, 24, 25).

The overlap with bvFTD includes frontal, temporal, and medial orbitofrontal cortex atrophy. As reported in older individuals with MCI (23), temporoparietal and superior frontal atrophy have been associated with poorer executive and visuospatial performance (13). The cerebellar volumetric changes, conventionally linked to aging, involved patterns across its transverse zones, corresponding to functional subdivisions characterized by distinctive cytoarchitectural and connectivity profiles (30).

The most common atrophy pattern in our young MMC cohorts is the cortical involvement of the parietal and frontoparietal lobes, combined with GM atrophy in cerebellar lobules IV and V (left) and III, IV, V, and VI (right).

PD-related atrophy patterns are also present (23, 31, 32). It is remarkable that the MRI striatal atrophy and significant caudate and putamen involvement, along with the hippocampus, thalamus, and amygdala atrophy, go hand in hand with forensic overlapping neurodegeneration and the subcellular abnormalities associated with combustion/friction and industrial NPs in MMC young residents (8–10). Degeneration of the nigrostriatal system occurs in multiple system atrophy (MSA) and PD (32). Strikingly, poorer cognitive performance among MMC residents was associated with globus pallidus and basal forebrain GM atrophy. The involvement of key subcortical structures in these clinically healthy young individuals with cognitive impairment is critical, as cognitive performance is strongly aligned with pallidal–thalamic–cerebellar integrity. Thalamic and deep GM nuclei are implicated in bvFTD (27), and seven bilateral subcortical structures, including the putamen, caudate nucleus, amygdala, hippocampus, thalamus, and globus pallidus, are involved in PD (33).

The importance of cortico-basal ganglia-thalamic (CBGT) circuits in young individuals exposed to pollution is substantial (34). Giossi et al. (34) support the concept that globus pallidus GM and WM serve as a central bidirectional hub within the CBGT circuit, playing a major role in behavioral regulation. The caudal part of the external globus pallidus is a major output nucleus of the auditory cortico-basal ganglia loop and has a pivotal role in eliciting defensive behaviors in response to acoustic stimuli, as described by Tomioka et al. (35). Moreover, alterations in the basal ganglia–dorsolateral prefrontal cortex (BG-DLPFC) network play a significant role in learning processes (36). Dorsal and ventral striatal signaling facilitate updating of choice behavior through thalamic pathways following both explicit-feedback and no-feedback trials (37).

In elderly populations, loneliness and cognitive decline have been linked to frontal WM atrophy, as well as putamen and globus pallidus alterations (38). Of particular concern is the association between initiation of substance use and targeted brain atrophy, an issue increasingly raised by MMC mental health professionals. Thinner prefrontal cortex has been associated with initiation of substance or alcohol use during pediatric ages (39). Psychopathy has been linked to atrophy in the pons, basal ganglia, thalamus, basal forebrain, cerebellum, and orbitofrontal, dorsolateral frontal, and insular cortices—regions significantly involved in MMC subjects (40).

Pieperhoff et al. (40) suggested that dysfunction in specific frontal-subcortical circuits, known to be critical for behavioral control, is strongly associated with antisocial behavior. This observation warrants investigation in northeastern and northwestern MMC areas characterized by high pollution and elevated homicide rates (3–6, 41).

BMI and cognitive trajectories are also major concerns for MMC residents, as cognitive deficits have been documented from childhood through early adulthood (11–14). Increased BMI has been associated with WM reductions involving the corpus callosum, bilateral posterior thalamic radiation, bilateral internal and external capsules, and bilateral inferior longitudinal and inferior fronto-occipital fasciculi (42). Herzog et al. (43) reported associations between higher BMI and poorer working memory, noting that dopamine-signaling single-nucleotide polymorphisms (e.g., Taq1A and DARPP-32), which influence striatal dopamine transmission, when combined with elevated BMI, shift the balance between distractor-resistant maintenance and updating in working memory tasks. Their findings (43) support both dopamine-dependent and dopamine-independent cognitive effects related to obesity, a major public health issue in Latin American cities (44). Lifelong weight changes are critical in evaluating cognitive health risk, and significant weight loss from midlife to late life has been linked to cognitive decline in older adults (45).

Cerebellar atrophy warrants specific consideration (46–50). We previously documented significant accumulation of magnetic NPs in the cerebellar cortex of MMC residents and their movement under 30–50 μT direct current magnetic field exposure (8, 10). The extensive cerebellar damage observed on MRI may be associated with ferrimagnetic NP movement under low magnetic fields, producing cytotoxic, hyperthermic, and free radical effects (9, 10). Gait and equilibrium abnormalities represent clinical correlates of cerebellar atrophy in MMC residents. Pierce and Péron (46) described abnormal responses to emotion recognition and reward valuation associated with cerebellar dysfunction, findings likely relevant to MMC residents. Hearing loss and decreased brainstem and cerebellar volumes in AD (47), as well as gait and cognitive abnormalities associated with regional cerebellar atrophy in elderly fallers (48), are also relevant for understanding risks in young urban populations.

Cerebellar involvement in amyotrophic lateral sclerosis (ALS) is particularly noteworthy. Tahedi et al. (49) described ALS cases with WM damage affecting thalamic projections to the anterior cingulate cortex, cerebellum, dorsolateral prefrontal cortex, Heschl’s gyrus, medial frontal gyrus, orbitofrontal cortex, parietal cortex, postcentral gyrus, and precentral gyrus. In the present study, precentral gyrus atrophy was documented in MMC residents, consistent with findings reported in ALS cases (49), along with cerebellar GM atrophy and extensive thalamo-cortical connectivity alterations. Tahedi et al. (49) proposed that degeneration of thalamic projections supports the *conceptualization* of ALS as a network disease. We support this view and extend it to major neurodegenerative diseases broadly, as TDP-43 pathology has been documented in association with AD and PD in MMC children and adolescents (8–10).

This study has two limitations. First, the sample size was limited due to budget constraints and safety concerns related to travel to certain regions in Mexico. Second, complex outdoor air pollution mixtures vary across MMC, and indoor pollution was not directly measured, although personal and household smoking were exclusion criteria. A major strength of this study is the ability to correlate neuroimaging findings with AD, PD, and TDP-43 forensic pathology in MMC children and young adults, alongside cumulative outdoor PM_2.5_ exposure data.

A key question remains: how much PM_2.5_ exposure is required to initiate neurodegenerative processes, structural brain changes, and cognitive deficits? Available evidence suggests that the threshold for neuropathological biological hallmarks is very low. An 11-month-old MMC infant demonstrated biological diagnoses consistent with AD and TDP-43 pathology after a total exposure of approximately 20 months, including in utero exposure, with cumulative PM_2.5_ levels above the annual US EPA standard of 20 μg/m^3^ (7). MMC residents exhibited a mean MoCA decline of 2.93 points over 20 years, indicating that MRI findings are not isolated and begin at pediatric ages (12, 13).

Our selected young middle-class cohorts, including adolescents without comorbidities, exemplify the profound environmental impact of longstanding PM_2.5_ air pollution in a megacity setting.

## 5 Conclusion

We must prioritize the neuroprotection of young populations. Prospective follow-up studies are urgently needed to determine the composition and size of PM pollutants impacting brain development, neurodegeneration, cognitive impairment, and neuropsychiatric disorders, and to explore early biomarkers of neurodegeneration. PM_2.5_ and UFPM measurements are essential, given that MRI-documented brain atrophy and cognitive deficits were observed in all examined MMC teens and young adults. Control of PM_2.5_ emissions, including those from heavy diesel vehicles, public transportation systems (e.g., the subway), and motorcycles, is urgently needed.

Hallmarks of AD, PD, FTLD, and ALS begin at pediatric ages and are progressive in the setting of PM_2.5_ pollution. Neuroradiologists worldwide should be aware of the early overlapping structural and volumetric MRI alterations.

## Data Availability

The original contributions presented in the study are included in the article/supplementary material, further inquiries can be directed to the corresponding author.
